# Expanding understanding of adolescent neural sensitivity to peers: Using social information processing theory to generate new lines of research

**DOI:** 10.1016/j.dcn.2024.101395

**Published:** 2024-05-24

**Authors:** Joseph S. Venticinque, Sarah J. McMillan, Amanda E. Guyer

**Affiliations:** a Center for Mind and Brain, University of California, Davis 267 Cousteau Pl, Davis, CA 95618, USA; b Department of Human Ecology, University of California, Davis 1 Shields Ave, Davis, CA 95616, USA

**Keywords:** Peer influence, FMRI, Adolescence, Individual differences, Behavior

## Abstract

Adolescence is a period of normative heightened sensitivity to peer influence. Individual differences in susceptibility to peers is related to individual differences in neural sensitivity, particularly in brain regions that support an increasingly greater orientation toward peers. Despite these empirically-established patterns, the more specific psychosocial and socio-cognitive factors associated with individual differences in neural sensitivity to peer influence are just beginning to gain research attention. Specific features of the factors that contribute to how adolescents process social information can inform understanding of the psychological and neurobiological processes involved in what renders adolescents to be more or less susceptible to peer influences. In this paper, we (1) review the literature about peer, family, and broader contextual influences on sensitivity to peers’ positive and negative behaviors, (2) outline components of social information processing theories, and (3) discuss features of these models from the perspectives and social cognitive development and social neuroscience. We identify gaps in the current literature that need to be addressed in order to gain a more comprehensive view of adolescent neural sensitivity to peer influence. We conclude by suggesting how future neuroimaging studies can adopt components of this social information processing model to generate new lines of research.

Humans are continually influenced by others around them. During adolescence specifically, peers are a primary agent of social influence, impacting one’s thoughts, feelings, and behaviors as well as neurophysiology (Guyer and Jarcho, 2018). Susceptibility to peer influence has been shown to peak during the adolescent years and remains elevated, albeit to a lesser extent, into young adulthood (Somerville, 2013, Steinberg and Monahan, 2007). Adolescents also spend significantly more time with their peers relative to parents (Ashbourne and Daly, 2012). Furthermore, adolescents seek out and participate in more complex and diverse social settings as they continue to form their own identities, thereby affording more opportunity for peer influence (Finkenauer et al., 2019, Mmari et al., 2021). Although heightened susceptibility to peer influence has traditionally been viewed as a unique hallmark of adolescence, there is evidence indicating that peers also continue to be a strong and salient source of influence on behavior and neurophysiology during young adulthood (Beard et al., 2022, Sherman et al., 2018, Venticinque et al., 2021). Informed by these developmental phenomena, a body of behavioral and neurobiological research has revealed a range of individual differences in adolescent susceptibility to peer influence (e.g., Markovitch and Knafo-Noam, 2021; Schriber and Guyer, 2016; Venticinque et al., 2021). As such, identifying various parameters and markers of susceptibility may help explain why some youth are more or less affected, in positive or negative ways, by peer influence.

One explanation for heightened susceptibility to peer influence during adolescence and young adulthood stems from maturational changes in how the brain processes and integrates peer-related social information. In adolescence, the brain is thought to undergo a social reorientation toward being particularly sensitive to peer input (Nelson et al., 2016). Thus, the confluence of increased neural “tuning” toward peers and dynamic shifts in social environments may render adolescents and young adults particularly sensitive to the influence of their peers, which in turn directs their behaviors. This is coupled with normative changes in pubertal development during adolescence, such that being more advanced in pubertal development relative to peers predicts greater risky decision-making, controlling for chronological age (Kretsch and Harden, 2014). Moreover, the widely reported behavioral effect of increased risk taking in the presence of peers among adolescents versus adults (Gardner and Steinberg, 2005) is attenuated by pubertal status, such that adolescents with more advanced pubertal development demonstrate less risky decision making in the presence of peers than when alone (Kretsch and Harden, 2014).

Typically guided by broad frameworks of normative adolescent brain development, such as social reorientation and dual systems models (e.g. Nelson et al., 2016; Shulman et al., 2016; Steinberg, 2008), much of the neuroscience research on peer influence has focused on how peer cues (e.g., presence of peers, peer evaluations) and external forces (e.g., peer group dynamics) influence affective and cognitive brain circuitry associated with risky decision-making (e.g., Chein et al., 2011; Telzer et al., 2015). In the behavioral developmental science literature, social information processing models (e.g., Beauchamp and Anderson, 2010; Crick and Dodge, 1994), have also delineated ways in which cognitive and socio-contextual factors influence how individuals process and respond to social information in their environments (e.g., Calvete et al., 2012), with a focus similar to the neuroscience literature on these factors’ contributions to positive and negative behavioral outcomes. Social information processing models may help to expand our understanding of neural sensitivity to peer influence during adolescence in several ways. Specifically, these models include a comprehensive range of psychosocial factors that impact neural sensitivity to peers, which have not yet been fully examined in neuroimaging research in this area. Additionally, social information processing models capture components of the underlying psychological and social constructs that neuroimaging tasks aim to simulate. Thus, these models may offer specific, measurable, and testable fundamental factors that extend and add value to existing models of adolescent brain development.

In the current review, we draw on social information processing models to examine a growing body of research focused on biopsychosocial factors that contribute to adolescent neural susceptibility to peer influence. First, we describe two relevant theoretical models of social information processing that have generated a considerable body of research relevant to peer influence: the social information processing model (SIP; Crick and Dodge, 1994) and the socio-cognitive integration of abilities model (SOCIAL; Beauchamp and Anderson, 2010). Second, using these social information processing models as a guide, we propose a conceptual framework to both interpret findings from existing peer influence neuroimaging studies and highlight gaps in our current understanding. Third, through the lens of our proposed framework, we review existing peer influence neuroimaging studies, which mainly focus on the contributions of the proximal social milieu and contextual factors (e.g., family relationships, peer characteristics, culture) that relate to peer-related processes (e.g., risky decision-making while being observed). Fourth, we review the socio-cognitive processes delineated within social-information processing models that have been not be well understood in the context of peer influence neuroimaging studies (e.g., cognitive processes related to how adolescents attend to, integrate, and respond to peer-related information). Finally, we conclude by suggesting links among these specific features of social information processing models to generate a more comprehensive understanding of youth neural sensitivity to peer influences.

The scope of the current conceptual review primarily includes studies that used functional magnetic resonance imaging (fMRI) as the primary measure to assess neural sensitivity to diverse forms of social influence, in samples of adolescents and young adults. Based on the available literature, we identified specific types of variables to cover in this review. Across studies, variables designated as predictors of neural susceptibility to social influence during adolescence and young adulthood include: experimentally-manipulated forms of influence (e.g., physical presence of peers, ratings provided by peers); self-reported forms of influence such as relationship quality with peers, and broader contextual factors that affect susceptibility to peer influence such as family, neighborhood, socioeconomic status, and cultural values. With regard to outcome variables resulting from susceptibility to peer influence, these include: changes in neural activity in relevant brain regions (e.g., those associated with reward-processing, salience detection, and social cognition) in response to task paradigms in which social information, primarily from peers, is delivered relative to other conditions or a neutral baseline; and behavioral measures such as performance on decision-making tasks, detection of socioemotional cues, and focus of attention tested via eye-gaze tracking.

## 1 The utility of applying social information processing frameworks

Advances in technology (e.g., neuroimaging techniques) have facilitated testing previously unanswered questions and forging new connections between the once disparate lines of research on peer influence and brain development during adolescence. Additionally, models of an interdisciplinary nature encourage hypotheses about how two or more processes work together, such as affective and cognitive processing). The simultaneous consideration of interactions that exist among characteristics of individuals, the contexts in which they are situated, and the processes that occur within those contexts more closely resemble the nuanced social milieu in which children and adolescents develop; for example, as espoused by Bronfenbrenner and Morris’ (2006) biopsychosocial model of development. Thus, in this review, we argue that adopting principles of social information processing models may help to expand the type of research questions being addressed in the peer influence neuroimaging literature. In this section, we will discuss two social information processing frameworks: (1) the social information processing model (Crick and Dodge, 1994) and (2) the socio-cognitive integration of abilities model (Beauchamp and Anderson, 2010) that, when applied to understanding how the brain responds to peer influence, may provide opportunities to incorporate previously unexamined factors into task paradigms used in neuroimaging as well as to account for contextual factors that influence specific components of social information processing.

### 1.1 Social information processing models

Crick and Dodge’s (1994) seminal social information processing (SIP) model was among the first to conceptualize how an individual integrates social information, processes the demands of the social context, and varies in adjustment to those demands. Crick and Dodge (1994) identified and proposed relations between specific information processing components that contributed to youth’s understanding of their social world and their behavioral output. In this model, several distinct stages are considered in response to a social situation: (1) sensory encoding of a social cue, (2) perceiving and integrating a social cue with a pre-existing knowledge base, (3) generating and implementing a response to the cue, and (4) evaluating the outcome. These steps were formulated as a sequential process by which youth would gather information from their social environment, reconcile it with their own existing experience of the world, cognitively devise a strategy to respond (or not respond) to the information, and then evaluate their response. Although not fully examined in their initial model, Crick and Dodge (1994) were among the first to examine how the biological factors a child or adolescent brings to their social interactions both shape and are shaped by their response to input from their social environment, primarily from peers during the adolescent years. Serving as a heuristic, one utility of the SIP model (and subsequent social information processing models) is that it serves to break down the subcomponents of how youth comprehend and respond to their social environments, an area not fully captured by the frameworks that have guided peer influence neuroimaging studies (e.g., Shulman et al., 2016).

Across several decades, the SIP model has been used as a guiding framework that generated a large body of research. For example, aggression among children has been thought to be linked to deviations in the outlined social information processing steps (Dodge, 2006). Specifically, children high in reactive aggression tend to interpret hostile intentions in the neutral actions of others, whereas children high in proactive aggression tend to expect positive rewards for being aggressive (Martinelli, 2018). Similarly, the processing steps outlined in the SIP model have been used to understand hostile attribution bias, in which individuals are more likely to interpret ambiguous situations as hostile than benign (Dodge et al., 2022, Epps and Kendall, 1995, Lansford et al., 2010). Overall, findings from this literature have shown that targeting the processing steps outlined by Crick and Dodge (1994) has been effective at changing behavior, for example, in treating aggressive behaviors among criminal offenders (Zajenkowska et al., 2021) and through interventions for youth with antisocial behaviors (Dodge and Godwin, 2013).

Expanding upon the SIP model, Beauchamp and Anderson (2010) proposed an integrative framework for understanding social skill development, known as the socio-cognitive integration of abilities model (SOCIAL). This model incorporates reciprocal interactions between mediators (e.g., external, and cognitive factors) and subsequent social functioning, defined in this framework as overall performance across many everyday domains (e.g., interpersonal relationships, autonomous living). Using a biopsychosocial approach, the SOCIAL model assumes that the emergence and trajectory of social functioning is dependent on normative maturation of the brain, cognition, and behavior. In other words, social functioning is mediated by the social brain network and is susceptible to environmental influences. In this model, external (i.e., contextual), and socio-cognitive (e.g., attention, socioemotional) factors interact bidirectionally with ongoing brain development to influence social behavioral outcomes. Unlike the SIP (Crick and Dodge, 1994), Beauchamp and Anderson (2010) further divided socio-cognitive constructs of their model into their subcomponents (e.g., attention/executive functioning). This model considers both typical and atypical development, such as changes in social functioning resultant from a traumatic brain injury. In this review, we will primarily focus on studies that examine normative adolescent development, in the context of the effects of peer influence. We draw upon studies of atypical development only to help further our understanding of typical development profiles and/or patterns.

### 1.2 Developing a conceptual framework incorporating components of the SOCIAL model

**Fig. 1:**
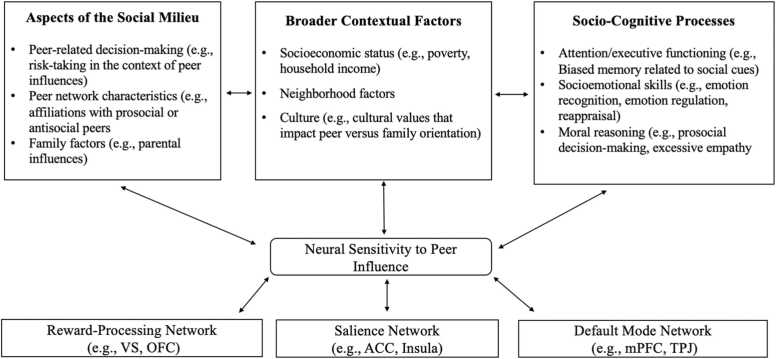
Conceptual Model of Applying Social Information Processing Theory to Investigate Neural Sensitivity to Peer Influence in Adolescence.

Beauchamp and Anderson (2010) outlined specific components of a biopsychosocial understanding of adolescent development and urged that components of their SOCIAL framework be operationalized and applied to specific bodies of research. To our knowledge, no literature review has applied the principles of the SOCIAL model to gain perspective on the biopsychosocial factors that shape how adolescents perceive, process, and respond—akin to that outlined in the SIP model—to peer influence. Thus, using the SOCIAL model as a working framework, we argue that an understanding of peer influence at behavioral and neural levels can be informed by incorporating components of social information processing. In this review, we propose a conceptual framework (see Fig. 1), guided by the core components of the SOCIAL model, and use this framework to evaluate existing peer influence neuroimaging studies and suggest areas that require more research attention. We selected the SOCIAL model as a primary framework (rather than directly applying the subcomponents of the SIP framework; Crick and Dodge, 1994) because, through building upon previous frameworks, it permits a comprehensive and testable view of the multi-level and bidirectional contributors to specific components of neural sensitivity to peer influence with respect to multiple levels of development.

Guided by Beauchamp and Anderson (2010), we have identified three broad categories of factors that impact neural sensitivity to peer influence among youth: (1) aspects of the social milieu (e.g., peer characteristics, family dynamics); (2) broader contextual factors (e.g., socioeconomic status, culture); and (3) socio-cognitive processes (e.g., attention, moral reasoning). The 18 reviewed studies within these categories (see Table 1) were assessed with regard to common patterns of activation of brain regions in three distinct networks: (1) the reward-processing network (e.g., ventral striatum, orbitofrontal cortex); (2) the salience network (e.g., insula, anterior cingulate cortex); and (3) the default mode network (e.g., medial prefrontal cortex, temporoparietal junction), as they are each implicated in social cognition (Spreng and Andrews-Hanna, 2015). It is important to note that these networks are also composed of other brain regions not identified in the reviewed work; further, these regions also support other functions beyond what is reviewed. To maintain an appropriate scope for the current review, we focus on specific functions and networks relevant for components of the included theoretical model. Studies were included if they involved fMRI tasks that targeted the constructs or behaviors within their respective sections of review. In the remainder of this review, we use our conceptual framework, guided by the SOCIAL model, as a lens to situate existing behavioral and neuroimaging literature related to sensitivity to peer influence, identify gaps in our understanding of these associations, and propose new lines of research to address these limitations.

**Table 1:** Summary of the 18 studies from the current review by category of influence, including fMRI tasks reflecting behaviors or decisions, activated brain regions within their associated networks, and relevant moderating factors.

Authors (Year)	fMRI task (s)	Activated Brain Regions	Brain Network (s)	Moderators
**Social Milieu**				
***Decision Making in Risky Contexts***				
Chein et al. (2011)	Stoplight task	VS, OFC	Reward	RPI
de Boer et al. (2017)	BART	NAcc	Reward	N/A
Telzer et al. (2015)	BART	VS, Insula, dlPFC	Reward, Salience, DMN	Peer support
***Peer Characteristics***				
Kim-Spoon et al. (2019)	Gambling lottery task	VS, mPFC	Reward, DMN	Cognitive control
Duell et al. (2021)	Prosocial decisions	Caudate, Insula, TPJ	Reward, Salience, DMN	Cortisol
Telzer et al. (2013)	Monetary gain task	VS, Insula	Reward, Salience	N/A
***Family Associations***				
Pozzi et al. (2020)	Emotion processing	OFC, Amygdala	Reward	N/A
van Hoorn et al. (2018)	Stoplight task	VS, dlPFC, TPJ	Reward, DMN	N/A
**Broader Contextual Factors**				
***Socioeconomic Status***				
Cascio et al. (2017)	Cyberball	Anterior Insula, dACC	Salience	SES
Gonzalez et al. (2015)	Cyberball	Right Insula, dACC	Salience	SES
***Culture***				
Chiao et al. (2009)	Self judgements	mPFC, Insula	Salience, DMN	Culture
**Socio-cognitive Processes**				
***Attention/Executive Functions***				
Oberwelland et al. (2017)	Eye-gaze task	TPJ	DMN	N/A
Oberwelland et al. (2016)	Joint attention task	TPJ, Precuneus	DMN N/A	
***Socioemotional Skills***				
Marusak et al. (2013)	Emotional faces	Amygdala, OFC	Reward	Emotion Type
Morningstar et al. (2018)	Emotional voices	Insula, TPJ	Salience, DMN	N/A
***Moral Reasoning***				
Sommer et al. (2014)	Moral dilemmas	mPFC	DMN	N/A
van Hoorn et al. (2016)	Monetary donations	mPFC, TPJ	DMN	Type of spectator
Tashjian et al. (2018)	Viewing prosocial scenes	TPJ	DMN	N/A

*Note:* OFC = orbitofrontal cortex; BART = balloon analogue risk task; RPI = resistance to peer influence; VS = ventral striatum; NAcc = nucleus accumbens; dlPFC = dorsolateral prefrontal cortex; mPFC = medial prefrontal cortex; DMN = default mode network; TPJ = temporoparietal junction; dACC = dorsal anterior cingulate cortex; SES = socioeconomic status; N/A = not applicable.

#### 1.2.1 Aspects of the social milieu

Many studies examining peer influence have focused on aspects of adolescent decision-making, often involving risk (e.g., effects of peer presence on simulated driving behaviors). Additionally, several of these investigations have centered on how external contextual factors (e.g., peer group dynamics) shape neural responses to peer influence, and how neural sensitivity shapes social interactions. In line with the first subcomponent of our framework (see Fig. 1), we review studies related to how aspects of the proximal social milieu of youth impact neural sensitivity to peer influence. We begin with discussion of neuroimaging studies of risk-based decision-making, as many of the reviewed studies under the social milieu category include having the youth make risk-based decisions in the presence of peers or decisions that impact peers negatively or positively. This focus in the literature likely reflects commonly held beliefs that adolescents have an inherent tendency to engage in risky behaviors, especially in the context of peers. Then, we describe studies examining aspects of the peer group (e.g., characteristics of the peers an individual affiliates with) and family relationship factors (e.g., decisions that may benefit a parent or a peer). Although the studies reviewed largely involve neuroimaging, we also draw upon behavioral findings where relevant.

### 1.3 Peer influence on decision-making in risky contexts

One of the core features of adolescence described in the behavioral and neuroimaging literatures is the propensity for heightened risk-taking, particularly in the presence of or as influenced by peers (Albert et al., 2013; van Duijvenvorrde et al., 2016). The dual-systems model (Shulman et al., 2016, Steinberg, 2008) sought to explain the neural bases underlying observed increases in risk-taking during adolescence, by positing that adolescent vulnerability to risky decision-making is largely in part due to the divergent developmental trajectories of two brain systems: an early-developing reward-driven system (e.g., ventral striatum, VS) and a later-developing cognitive control system (e.g., lateral prefrontal cortex) (Steinberg, 2008). Due to this mismatch in neurodevelopmental timing, it is thought that adolescents tend to seek out and engage in highly appetitive (sometimes reckless) behaviors, without the proper cognitive-control circuitry to curb impulsive behaviors. This intuitive perspective has generated a large body of work aimed at testing the role of peers in sensitizing the brain to risky decision-making during adolescence. A primary focus of this research has been on how the presence (or perceived presence) of socializing agents, primarily peers, impacts the neural circuitry involved in how adolescents make decisions involving risk.

One commonly-used fMRI task for examining risky decision-making is Stoplight, a simulated driving task in which adolescents “drive” through a series of yellow lights, quickly deciding whether to speed through or come to a stop. In this context, adolescents show increases in both behavioral risk-taking (represented by more crashes) and activation in reward-processing brain regions (e.g., VS; orbitofrontal cortex, OFC) on trials when they believe a peer is watching, compared to trials without observation, a pattern not found in adults over the age of 25 (Chein et al., 2011). Similarly, in another task, adolescents display increased neural activation in reward-responsive brain regions (e.g., nucleus accumbens, NAcc) when they believe they were being viewed by a peer while inflating a virtual balloon for increasing monetary rewards versus trials without observation from peers (de Boer et al., 2017). Another line of research has examined how adolescents’ perceptions of the quality of their peer relationships influences neural sensitivity when making decisions involving risk. For example, higher levels of peer conflict have been associated with greater risk-taking behaviors and activity in brain regions in the reward-processing network (e.g., VS), salience-detection network (e.g., bilateral insula), and the dorsolateral prefrontal cortex (dlPFC), functionally connected to the default mode network (Ahuja and Rodriguez, 2022) when adolescents were making decisions involving risk; and specifically for adolescents who reported low peer support (Telzer et al., 2015). In other words, feeling a lack of support from peers may confer vulnerability to risky behavior via neural mechanisms involving reward sensitization to quality of peer experiences. At the same time, it is possible that adolescents who tend to respond more strongly to rewards could experience more conflict with their peers, such that there is a bidirectional link between neural reactivity and social functioning. Still, these patterns suggest that having a more supportive peer group may mitigate the negative effects of peer conflict and increased risk-taking. Taken together, these studies highlight the powerful influence of the presence of (or relationship with) peers on engagement of reward-responsive brain regions when adolescents make decisions involving risk.

### 1.4 Peer characteristics

Considering the characteristics of adolescents’ peers and peer group is crucial to understanding individual differences in neural sensitivity to peer influence. For example, variability in the behaviors of peers that adolescents affiliate with (e.g., how often their peers engage in deviant or positive behaviors) may impact how adolescents process and respond to peer influence; and neural reactivity to peer influence could be a factor that shapes selection into specific peer groups. A body of research has examined how affiliating with deviant peers confers vulnerability for adolescents to be more sensitive to risk-taking via neural mechanisms (e.g., Albert and Steinberg, 2011; Chein, 2015; Kim-Spoon et al., 2019). For example, using a gambling lottery task, Kim-Spoon and colleagues (2019) found that adolescents whose peers engaged in high versus low substance use behaviors displayed greater activity in brain regions implicated in risk-taking (e.g., VS). Conversely, less activity was displayed in cognitive control brain areas (e.g., medial prefrontal cortex, mPFC) during this task. These findings revealed a brain-behavior association that can help identify adolescents who may be vulnerable to peer contagions, and are consistent with results from behavioral studies demonstrating that affiliating with deviant peers relates to one’s own delinquency through both peer selection and peer socialization processes that increase one’s similarity to peers (Monahan et al., 2009, Prinstein et al., 2011). These effects appear to be present across middle and late adolescence, but diminish after age 20, suggesting that adolescence is a sensitive period for conforming to deviant peer behaviors (Monahan et al., 2009).

Other work has been conducted with a goal of reframing the stereotype that increased time with peers during adolescence puts individuals at risk for negative behaviors, by testing whether certain peer characteristics may be leveraged to promote positive youth development (Telzer et al., 2018). In other words, affiliating with positive peers (i.e., who tend to engage in prosocial behaviors) may buffer adolescents from some of the reported negative effects of peer influence or promote positive outcomes (Beard and Wolff, 2022), namely by attenuating activity in the reward-processing brain regions that are particularly engaged by peer stimuli during this developmental period. For example, within the context of an fMRI task, adolescents were exposed to peers who were either low or high in prosocial behaviors, and their conformity to peers’ prosocial donation behaviors was measured (Duell et al., 2021). Of note, greater conformity to high vs. low prosocial peers’ donation behaviors was related to greater activation in brain regions involved in social cognition (e.g., temporoparietal junction [TPJ]), salience detection (e.g., insula), and reward processing (e.g., caudate). These findings highlight that while peers are salient influencers on the brain, distinguishing between the types of behaviors that an adolescent’s peers engage in revealed differential responses in specific brain regions. Furthermore, a history of engaging with prosocial peers has been shown to predict diminished sensitivity in the VS during risk-taking (Telzer et al., 2013). In line with the SOCIAL model, this association could also be bidirectional, such that adolescents who demonstrate less reward reactivity tend to select more prosocial peers as friends.

### 1.5 Family associations with susceptibility to peer influence

Adolescents undergo a well-established social reorientation toward peers during adolescence through exploring one’s identity and establishing autonomy by growing peer networks beyond the family unit (Nelson, et al., 2016). Nonetheless, family members (primarily parents/caregivers) remain important agents of socialization. Adolescents must reconcile how much (and in what contexts) they rely on parents as the primary source of guidance, versus finding solace in their newly developing peer relationships (Fabes et al., 2009). Thus, understanding both the unique and shared contributions of peers versus family members to how an adolescent’s brain processes social information has been an area of growing interest. Similarly, parental and peer influences have been shown to have differential effects on other physiological systems, such as the hypothalamic-pituitary-adrenocortical (HPA) axis stress response (Gunnar and Hostinar, 2015, Hostinar et al., 2015).

Parents and peers both play an important role in shaping how adolescents learn, internalize, and form preferences, yet little is known about how the influence from these various socialization sources interact and together shape the underlying cognitive processes of social influence across development. Behavioral work has created experimental scenarios in which adolescents must consider both of these sources of influence (i.e., from a parent or peer) in juxtaposition to each other. For example, Guassi Moreira and colleagues (2018) used a behavioral modification of the Columbia Card Task to test how young adults made gambling decisions that benefitted either a peer or parent, at the direct detriment to the relationship that they did not choose. Results from this study showed that young adults were more likely to make decisions that benefited a parent, at the expense of a friend, than vice versa. This effect was moderated by adolescent-reported relationship quality with parents and friends, indicating that high relationship quality with either close other was associated with prioritizing that close other during decision-making. Of note, it is unknown whether the reverse effect might be observed for adolescents, leaving an unanswered yet important developmental question. Furthermore, much of the extant research that has examined parental influences on the adolescent and young adult brain has focused solely on influence from mothers (e.g., Gee et al., 2014; Guassi Moreira et al., 2018; Pozzi et al., 2020). More work is needed to understand whether influence from fathers or other primary caregivers elicits similar or different neural profiles relative to maternal influence for adolescents.

In regard to risk-taking, van Hoorn and colleagues (2018) sought to disentangle the differential effects of parental (majority mothers but including fathers) and peer influence on neural sensitivity when adolescents are engaged in risky behaviors, whereby influence was operationalized as the physical presence of each socialization agent. Results indicated differential neural profiles, such that brain regions involved in reward response (e.g., VS) and socio-cognitive response (e.g., dlPFC) were more active in the presence of peers than the presence of parents during a simulated driving game (in line with findings from Chein et al., 2011). However, functional connectivity analyses revealed greater coupling between reward processing and socio-cognitive brain regions in the presence of parents versus peers (van Hoorn et al., 2018). These findings suggest that peer presence elicits overall more engagement of reward-related and socio-cognitive brain regions independently during risky decision-making, but parental presence relates to more effective co-activation of these regions, possibly reflecting adaptive regulation of one’s behavior.

## 2 Broader contextual factors

### 2.1 Socioeconomic status

Moving beyond the proximal social worlds of youth to more distal contexts, socioeconomic status (SES) of adolescents and their families, as well as that of the neighborhood/community within which they reside, are also relevant factors to consider in examining adolescent susceptibility to peer influence. Hyde and colleagues (2020) describe these processes as a “neural embedding of poverty” in which the adolescent brain is a mediator of how youth adapt to stressful contexts. The authors describe that this process is most saliently transmitted via proximal (e.g., peer interactions) rather than distal (e.g., government policies) influences. Similarly, some have argued that poverty may tune the brain to pay more attention to salient cues in order to avoid threats, and rely more heavily on interpersonal relationships such as one’s peers to cope with adversity (Varnum and Kitayama, 2017). Thus, through specific neural underpinnings, adolescents in poverty may be particularly attuned to peers as a support mechanism to cope with stressful, often unpredictable environments, bringing attention to the need to consider the influence of SES and broader contextual factors on adolescent neural sensitivity to peer influence.

Specifically examining the role of SES in neural sensitivity to peer influence in adolescence, Cascio and colleagues (2017) tested whether SES moderated the association between brain responses to peer influence and behavioral performance on a simulated driving task. Results indicated that SES moderated this link, whereby increased activity in brain regions implicated in social pain and reward sensitivity during peer influence was associated with greater conformity to peers’ propensity for risk-taking among adolescents of low SES backgrounds; and decreased conformity among those of high SES backgrounds. Similarly, evidence from Gonzales and colleagues (2015) indicates that adolescent girls from lower SES backgrounds display greater activation in brain regions involved with social pain (e.g., dorsal anterior cingulate cortex, dACC) during social exclusion from peers, relative to more affluent adolescents. This suggests that peer influence when operating as a social threat may be more salient for adolescents from low SES backgrounds. Interventions aimed at improving SES (e.g., Noble et al., 2021; Troller-Renfree et al., 2022) may therefore have residual benefits for neural circuitry (e.g., regulating emotions and dampening heightened reactivity to rewards) that is implicated in adolescent peer relationships and peer influence.

Much of the extant literature examining the role of SES on adolescent brain development has been framed from a risk perspective examining the potential detriments of growing up in a low-income family, with little known about the potential buffers, supports, or unique challenges faced by adolescents from higher income backgrounds, or adolescents whose families experience improvements in income. For example, a study examining salivary cortisol responses to social stress among youth who experienced chronic poverty found that youth whose household income worsened over adolescence displayed an elevated overall stress response and recovered less quickly from acute stress (Johnson et al., 2021). This suggests that the stress response system to social stress appears to continue to be malleable throughout adolescence, and increasing household income among impoverished youth may promote healthy functioning. However, this has yet to be examined at the level of the brain using neuroimaging techniques in relation to peer influence during adolescence.

### 2.2 Culture

Further expanding to more distal contexts of development (Bronfenbrenner and Morris, 2006), the effects of cultural factors must be considered for explaining variation in behavioral and neurobiological susceptibility to peer influence. Adolescent susceptibility to peer influence has been examined cross-culturally in the behavioral literature, with findings suggesting differences in peer susceptibility between individualistic and collectivist cultures (Yang and Laroche, 2011). For example, differences in parental socialization goals between families in Canada and China moderated adolescent susceptibility to peer influence, such that responsive parenting in a more collectivist culture had a greater direct effect on reducing peer influence susceptibility than in an individualistic culture (Yang and Laroche, 2011). In a sample of culturally diverse Australian adolescents, higher rates of cultural identity (e.g., actively participating in groups of activities related to one’s culture) were found to be protective by reducing susceptibility to peer influence related to engaging in substance use (Gazis et al., 2010). Similarly, peer influence on smoking behaviors differs based on ethnic/racial factors, with White adolescents shown to be more susceptible to friends’ smoking behaviors than several minority groups with ethnic backgrounds from more collectivist cultures, such as Hispanic populations (Unger et al., 2001). Findings from these studies highlight the role that cultural factors play in heightening or potentially buffering susceptibility to peer influence in adolescence. However, more behavioral and neurobiological work is needed to identify specific—rather than broad—aspects of culture and ethnicity that may confer risk for or buffer from negative peer influence, and that considers promotive effects from positive peer influence. For example, designing a task paradigm that considers Latino cultural values related to avoiding conflict (i.e., simpatia) and having strong orientation to prioritizing family (i.e., familismo) may provide insight into unique within culture differences in peer influence susceptibility.

With regard to examining the role of the brain, cultural neuroscience seeks to understand how socio-cultural level factors influence neural processing (Ames and Fiske, 2010, Chiao et al., 2013). Many cultural neuroscience studies have examined brain-based differences among various ethnic and racial groups (e.g., Harada et al., 2020; Kroon et al., 2023), but additional neuroimaging work is needed to examine cross-cultural differences and specific within-culture effects that may further contribute to variation in neural sensitivity to peer influence among adolescents from different ethnic groups. Moreover, future research can identify mechanisms of cultural influence on brain function and social influence. Effects of culture have often been challenging to disentangle from other factors, such as SES, with many studies using proxies for culture without directly measuring cultural features. For example, the previously reviewed study from Cascio and colleagues (2017) reports culture as a moderating link between peer influence and behavioral risk-taking; however, SES level was included in lieu of measuring cultural factors directly. In recent years, there has been an effort to test questions of neural processing beyond using White, educated, industrialized, rich, democratic (WEIRD) samples (Burns et al., 2019, Chiao and Cheon, 2010). However, there remains a paucity of neuroimaging studies in the peer influence literature that consider cultural factors or draw participants from non-WEIRD samples.

## 3 Socio-cognitive processes

Much of the existing peer influence neuroimaging literature has focused primarily on how aspects of the social environment or contextual factors contribute to shaping adolescent neural responses to peers (reviewed in the previous sections of this paper), without consideration for the underlying cognitive processes involved in integrating that social information from the environment that then feeds into neural response and behavioral output. Gaining a better understanding of how adolescents are cognitively processing the stimuli presented to them in fMRI tasks has important implications for how neuroimaging findings can be interpreted and applied to real-world social contexts. Aligning with the socio-cognitive subcomponent of our framework, guided by Beauchamp and Anderson (2010), we next discuss studies examining how cognitive processing relates to observed neural responses to peer influence, an area that has not been thoroughly considered in the available literature. First, we review studies focused on attention and executive functioning. Next, we review studies that focus on socio-emotional skills (e.g., emotion recognition) and moral reasoning. We aim to highlight links between brain regions implicated in socio-cognitive studies and the external and contextual subcomponents of the SOCIAL model. Due to the limited number of neuroimaging studies in this area, we draw upon behavioral studies where relevant.

### 3.1 Attention/executive functions

Accounting for attention and executive functioning is central to understanding how adolescents evaluate and respond to peer influence. Methods devised to measure aspects of social interactions to which adolescents attend have informed neuroimaging studies by highlighting the types of cues that are most salient. Eye-tracking, and its associated metrics such as gaze duration and arousal, is a useful behavioral technique that provides information about what information individuals pay attention to (Shagass et al., 1976). For example, using an interactive *Chatroom Task* in which adolescents received rejection and acceptance feedback from virtual peers, Silk and colleagues (2012) measured pupillary dilation, a behavioral correlate of increased activity of cognitive and affective brain regions, when adolescents experienced peer rejection. Results indicated an attentional bias toward positive feedback from peers and away from rejection feedback, with adolescents appearing to anticipate and avoid rejection feedback; and that the salience, measured through pupillary response, of this phenomenon increased with age.

Although considering attentional biases has utility when studying various groups of individuals, it has often been used to uncover differences among atypically developing youth; and comparisons among these populations contribute to our understanding of typical development as well. For example, these techniques can be particularly useful for characterizing the social interactions of adolescents with disorders involving impairments in social communication such as Autism Spectrum Disorder (ASD) that can alter the ways in which autistic adolescents process social-emotional cues (Wagner et al., 2013). Similarly, eye gaze behaviors have been measured among children and adolescents with specific language impairment (SLI) while they engage in social interactions with their peers (Hosozawa et al., 2012), revealing similarities and differences among both typically developing adolescents and autistic adolescents. Thus, considering attention when examining peer interactions and differences in susceptibility to peers is crucial for revealing nuance among both typically and atypically developing adolescents. Finally, related to risk-taking, adolescents have been shown to exhibit increased pupillary dilation when making risk-based decisions, relative to adults (Rosenbaum et al., 2021). Future studies should attempt to incorporate these measures when presenting peer-related stimuli to participants as they have the potential to generate new findings that can enhance understanding of how peer influence manifests and affects typical and atypical behavior.

Technological advances have facilitated the tracking of eye movements while collecting fMRI data simultaneously, providing multimodal information about how adolescents process social information (Pfeiffer et al., 2013). For example, a gaze contingent fMRI study of autistic youth reported differences in eye-gaze behaviors and uncovered abnormal activation patterns of brain regions in the joint attention network (e.g., superior temporal sulcus, TPJ; Oberwelland et al., 2017). In another study combining eye-tracking and fMRI, Oberwelland and colleagues (2016) demonstrated that the TPJ and precuneus are activated and play a crucial role in initiating and maintaining joint-attention with a social partner. Others have also suggested that using a combination of eye-tacking and fMRI may be useful for other groups of adolescents such as those diagnosed with social anxiety disorder (Capriola-Hall et al., 2020). Although there is some limited evidence to suggest that these reviewed studies demonstrate overlap in brain regions of the default mode network (e.g., TPJ) with studies involving socio-contextual contributors to neural sensitivity to peer influence previously reviewed (e.g., Duell et al., 2021), more work is needed to directly link aspects of attention/executive functioning to peer influence fMRI task paradigms. Given the reviewed literature, it is clear that supplementing fMRI tasks of peer influence with eye-tracking measures has the potential to reveal new information about what aspects of peer-related stimuli adolescents attend to, and to help identify individuals at risk for negative outcomes or in need of intervention.

### 3.2 Socio-emotional

According to the Beauchamp and Anderson (2010) SOCIAL model, socio-emotional dimensions include the ability to detect differences in facial affect and prosody (i.e., pitch, intensity, emotional content of voices), and higher-level cognitive processes such as perspective taking. These processes are described as being hierarchical, beginning with basic emotion recognition and then extending into more complex processes, such as understanding others’ mental states. Effectively perceiving, identifying, and responding to emotions in facial expressions is central to effective social reciprocity (McClure, 2000). Furthermore, being able to recognize and understand the emotions of others, particularly peers, has been demonstrated to influence how adolescents react to and adjust their own behaviors in social settings. For example, greater accuracy in identifying peer emotional expressions has been linked to lower rates of engaging in bullying behaviors (Pozzoli et al., 2017). Effective recognition of others’ emotional states has been shown to be modulated by age, gender, and pubertal status, with linear increases found across development and with girls showing greater effectiveness sooner relative to boys (Lawrence et al., 2015).

A longstanding line of neuroimaging research has examined how individuals perceive and respond to various emotions expressed by faces (e.g., Guyer et al., 2008; Jehna et al., 2011; Kesler et al., 2001; Narumoto et al., 2001). When comparing adolescent neural responses to emotional facial expressions of both adults and other adolescents, there appears to be age-selective salience of certain emotions. Specifically, in one study, adolescents displayed the strongest amygdala response to presentations of negative emotions in adults, but conversely the lowest to positive facial expressions among youth actors (Marusak et al., 2013). This suggests that adolescents may be particularly cued-in to expressions of negative emotions from parental figures (possibly in the context of discipline), but also more cued-in to positive expressions from peers as a method of socialization.

Recent studies have also begun to examine how emotional content in peers’ voices is processed by the adolescent brain. High emotional intelligence has been found to relate to increased ability to integrate emotion recognition cues across various modalities during adolescence (e.g., face and voice; Davis et al., 2020). Some evidence also suggests that vocal emotion recognition follows a protracted developmental trajectory, relative to facial emotion recognition with effective recognition of emotional voices coming online later during adolescence, evidenced by changes in nodes of the salience (e.g., insula) and default mode (TPJ) networks (Morningstar et al., 2018). Effective recognition of emotion via voice is an important skill that intersects with peer influence, as understanding the tone of voice of a peer provides information about peers’ potential motivations or emotional states. There remains a paucity of behavioral or neuroimaging research examining longitudinal changes in recognizing vocal emotions cues during adolescence, with most existing studies comparing cross-sectional samples of youth to adult listeners. Some researchers have begun to develop task stimuli that incorporate facial and vocal emotional content simultaneously examining how adolescents process these two modalities in conjunction (Liu et al., 2012). Uncovering the combined influence of these sources of emotional information (particularly if they provide conflicting information) in neuroimaging research is crucial to better understanding how adolescents process and interpret others’ emotions, a skill involved in understanding and responding to peer influence.

### 3.3 Moral reasoning

Moral reasoning broadly relates to how individuals make decisions regarding right and wrong (Wainryb, 2004). This construct has been closely linked to having what is called theory of mind (Wellman, 1990), in which being able to understand and interpret the mental states of others is central; but moral reasoning more closely considers the intention and justification underlying moral actions (Astington, 2004). These social-cognitive abilities have been supported by a neuroimaging study in which adolescents were presented with moral conflict scenarios, and showed high engagement of brain regions that are centrally involved in theory of mind (e.g., medial prefrontal cortex) when making morally-oriented decisions (Sommer et al., 2014). Applied to the peer influence literature, researchers have designed tasks or scenarios in which adolescents must use moral reasoning influenced by peers to make decisions that benefit themselves or others. For example, in a public goods donation fMRI paradigm, in which adolescents made decisions about allocating tokens to themselves and a group either alone or while being observed by age-matched peers, peer presence resulted in increased neural activity in the medial prefrontal cortex, TPJ, and superior temporal sulcus when adolescents allocated tokens to the group (van Hoorn et al., 2016). These regions comprise the default mode network, involved in social cognition; and their involvement suggests that the enhanced sensitivity to peer influence may also extend to positive forms of decision-making in adolescence. Additionally, adolescents have displayed increased TPJ activity when viewing scenes in which peers engage in prosocial donations versus either neutrally-social or noninteractive scenes (Tashjian et al., 2018). These findings differed based on an adolescent’s own propensity to engage in charitable giving behaviors, implying that adolescents who show more prosocial tendencies could be more tuned in to peers’ prosocial behaviors. Given that peers can influence prosocial behaviors, this pattern suggests a potential transactional link between prosociality and neural response to prosocial scenes. Thus, adolescence may be a window of opportunity to expose adolescents to prosocial decision-making, which has implications for leveraging the power of peers to encourage positive moral reasoning skills during this period of heightened sensitivity to peers.

Taken together, it is apparent that greater attention is needed in research toward the various socio-cognitive processes that underlie peer influence processes, in both behavioral and neuroimaging studies, in order to uncover possible mechanisms contributing to individual differences in sensitivity to the influence of peers. Relatively little work has bridged the gap between developmental cognitive and social neuroscience, yet doing so can clarify how adolescents process the flood of peer-related information present in their everyday environments. There is limited, but growing evidence of overlap among brain regions in the salience, reward, and default mode networks from studies of socio-cognitive processes and socio-contextual studies that are prevalent in the peer influence neuroimaging literature. Cultural neuroscience can also be applied to reflect differences in values about the role of family or peers, in order to deepen our understanding of cognitive processes within social contexts. In line with the SOCIAL model, more work is needed to specifically test how the subcomponents of cognitive processing may modulate neural susceptibility to peer influence during adolescence.

## 4 Conclusion

Based on our summary of the reviewed studies, it is clear that applying social information processing frameworks has the potential to explain findings from the peer influence neuroimaging literature and identify gaps in our current understanding. Specifically, much of the existing literature has focused on factors of individual’s proximal social milieu (e.g., peer group characteristics) and broader contextual factors (e.g., SES) that affect susceptibility to peer influence, with less attention focused on the socio-cognitive processes underlying how typically developing adolescents perceive and integrate peer cues from their environments. Additionally, there has been little focus on the possible bidirectional associations among social behaviors, socio-cognitive processing, and neural sensitivity to social and rewarding situations. Further attention to these socio-cognitive processes, as guided by social information processing models, will be important for gaining insight into the particular aspects of peer influence that adolescents may be attending to and how they make decisions given different types of inputs from peers. What has been missing from this line of research, is a focus on what specific social information processing steps are involved in how peer presence influences adolescents such as what information about the peer they encode, what cues they perceive, attend to and integrate with their existing knowledge, how they respond to the peer cues, and how they evaluate the outcome of the peer’s presence.

The reviewed studies of socio-contextual factors contributing to neural sensitivity to peer influence revealed patterns of common activation in brain regions of the salience, reward, and default mode networks. Limited evidence has shown potential transactional links between socio-cognitive processes, socio-contextual factors, and activation in these three identified brain networks. For example, nodes of the reward network (e.g., VS, caudate) are implicated in not only peer influence on risk-taking (e.g., Kim-Spoon et al., 2019), but also conformity to peers’ prosocial behaviors (Duell et al., 2021). This conformity task also elicited activity in the insula, a key node of the salience network; and other work has revealed greater insular activity while taking risks among adolescents who experience high conflict with peers. Regions of the default mode network – including the TPJ, STS, mPFC, and dlPFC – are involved in several socio-cognitive and socio-contextual processes. Activity in the TPJ has been linked to joint attention with a social partner (Oberwelland et al., 2016), prosocial conformity (Duell et al., 2021), prosocial behaviors when being observed by peers (van Hoorn et al., 2016), and observation of prosocial behaviors in others (Tashjian et al., 2018). Beyond typically developing youth, altered TPJ activity has also been identified among autistic adolescents (Oberwelland et al., 2017), along with altered STS activity. Prior work also found STS response while engaging in prosocial behaviors (van Hoorn et al., 2016). Lastly, the mPFC has been implicated in reward processes and peer influences (Kim-Spoon et al., 2019), but also moral conflict (Sommer et al., 2014) and prosocial behaviors (van Hoorn et al., 2016); and dlPFC to reward has been associated with peer conflict (Ahuja and Rodriguez, 2022). Taken together, the neuroimaging literature suggests substantial overlap between social influences and activation of nodes in the salience, reward, and default mode networks. Many of the reviewed studies included testing brain regions within one or two of these identified networks. However, future peer influence neuroimaging studies that consider nodes of all three networks, and their associated psychological and behavioral correlates, are needed to more comprehensively understand neural sensitivity to peer influence.

Due to relatively few longitudinal neuroimaging designs, the direction of the described relations among neural sensitivity, socio-cognitive factors, and socio-contextual factors remains unclear. Much of the existing work has tested how aspects of an individual’s socio-contextual environment predict concurrent or subsequent neural sensitivity to peer influence, but understanding the transactional relations of how neural sensitivity to peers impacts the ways that adolescents later think and behave warrants further research. For example, future work should focus on the bidirectional links among social-contextual environments (family, peers, schools, communities), adolescent behaviors (risk-taking, conformity, social decision-making), and neural sensitivity to peers and other agents of social influence. Better understanding the interplay between these once disparate lines of research will provide a more comprehensive understanding of how youths’ social environments, behaviors, and neurobiology interact in the context of peers.

Neural sensitivity to peer influence in adolescence is a complex phenomenon shaped by a host of biological, contextual, and cognitive factors. Much of the existing peer influence neuroimaging literature has been guided by broad frameworks of normative brain development (Nelson et al., 2005; Steinberg, 2008). Although these frameworks have profoundly advanced our understanding of normative adolescent brain development, they lack the specificity to be rigorously tested and thus have yielded inconsistent results. Using the SOCIAL model as a guiding framework, future studies can reveal possible bidirectional links among social functioning, neural sensitivity, and moderators and mediators such as SES. Future work is also needed to develop valid and reliable measures to operationalize the factors that influence the ways that adolescents perceive, integrate, and respond to peer influence cues in their everyday social environments. In conclusion, integrating aspects of social information processing models (Beauchamp and Anderson, 2010, Crick and Dodge, 1994) within new research questions has the potential to reveal information about the contributing factors to neural sensitivity to peer influence in adolescence, namely in expanding understanding of underlying cognitive processes and their interaction with external factors such as family relationships. These goals have the potential to move the field toward a more comprehensive understanding of how adolescents perceive, process, and respond to peer influence, which in turn may help inform interventions or preventative measures designed to support adaptive functioning in adolescence.

## Data statement

Availability of data not applicable for the submitted literature review.

## CRediT authorship contribution statement

**Amanda E. Guyer:** Writing – review & editing, Supervision, Conceptualization. **Sarah J. McMillan:** Writing – review & editing, Conceptualization. **Joseph S. Venticinque:** Writing – original draft, Methodology, Conceptualization.

## Declaration of Competing Interest

The authors declare that they have no known competing financial interests or personal relationships that could have appeared to influence the work reported in this paper.

## Data Availability

No data was used for the research described in the article.
